# Unlearned visual preferences for the head region in domestic chicks

**DOI:** 10.1371/journal.pone.0222079

**Published:** 2019-09-03

**Authors:** Orsola Rosa-Salva, Uwe Mayer, Giorgio Vallortigara

**Affiliations:** Center for Mind/Brain Sciences, University of Trento, Rovereto (TN), Italy; University of New England, Australia, AUSTRALIA

## Abstract

Unlearned tendencies to approach animate creatures are of great adaptive value, especially for nidifugous social birds that need to react to the presence of potential social companions shortly after hatching. Domestic chicks’ preferences for taxidermized hens provided the first evidence of social predispositions. However, the nature of the stimuli eliciting this predisposition is not completely understood. Here we explore the unlearned preferences of visually naïve domestic chicks for taxidermized animals. Visually naive chicks were tested for their approach preferences between a target stimulus (an intact stuffed animal whose head region was clearly visible) and a control stimulus. After confirming the predisposition for the intact stuffed fowl hen (Exp. 1), we found an analogous preference for a taxidermized, young domestic chick over a severely scrambled version of the same stimulus, whose body structure was completely disrupted, extending to same-age individuals the results that had been obtained with taxidermized hens (Exp. 2). We also directly tested preferences for specimens whose head region is visible compared to ones whose head region was occluded. To clarify whether chicks are sensitive to species-specific information, we employed specimens of female mallard ducks and of a mammalian predator, the polecat. Chicks showed a preference for the duck stimulus whose wings have been covered over a similar stimulus whose head region has been covered, providing direct evidence that the visibility of the head region of taxidermized models drive chicks’ behaviour in this test, and that the attraction for the head region indeed extends to females of other bird species (Exp. 3). However, no similar preference was obtained with the polecat stimuli (Exp. 4). We thus confirmed the presence of unlearned visual preferences for the head region in newly-hatched chicks, though other factors can limit the species-generality of the phenomenon.

## Introduction

The ability to detect animate creatures is of great adaptive value for most animals, since it allows the identification of potential preys or predators, as well as conspecifics, i.e. potential social companions and competitors [1,2]. Early social predispositions are unlearned tendencies to approach or attend to stimuli presenting features that are typical of animate creatures, which can be found already in very young organisms (reviewed in [3,4]). Similar social predispositions have been observed in different social species, including non-human primates [5], human newborns [6] and visually naïve newly-hatched domestic chicks [7,8], indicating the presence of mechanisms of great adaptive value, which do not depend on previous experience of the preferred animals. Indeed, the role of social predispositions in newborn organisms could be not only to promote social bonding with conspecifics, but also to ensure opportunities to learn about the features of appropriate social partners, by increasing exposure to them during sensitive periods in the ontogenesis [9,10]. This is particularly important for the development of brain circuits specialised in processing social stimuli. Anomalies of the social predispositions could thus lead to atypical development of the adult social brain, with potentially dramatic consequences for social adaptation, as seen in autistic spectrum disorders [6,11–13].

Recent research has revealed the presence of social predispositions directed to dynamic stimuli, whose motion resembles that of animate creatures (e.g. [14–21]). However, the first evidence of social predispositions did not regard the motion properties of the stimuli, but rather the static configuration of figures that characterize the face region of most animals. In a seminal series of studies from the research group then lead by Gabriel Horn, visually naïve domestic chicks were found to have an emerging preference for approaching a taxidermized red junglefowl hen, compared to artificial stimuli, such as an illuminated red box (e.g., [7]). This predisposition has been explored in multiple studies, clarifying its temporal features, the role of non-specific stimulating experience in its development, and some of its physiological correlates (e.g. [22–26]). Domestic chicks represent an optimal model for the investigation of social predispositions, since they can be tested directly after hatching in a dark incubator, allowing the detection of early preferences for social stimuli, which cannot be attributed to early learning from visual experience occurring before the test moment [27]. Moreover, chicks, like other precocial nidifugous birds, are capable of filial imprinting, a mechanism of learning by exposure that typically restricts their affiliative responses to the first conspicuous object(s) the hatchlings are exposed to [28–33]. One of the adaptive functions of social predispositions could be precisely to direct filial imprinting towards a plausible object. By directing the animals’ attention towards animate creatures, a social predisposition may facilitate imprinting towards an appropriate social object (i.e. the mother hen or another chick of the brood). However, social predispositions are supported by very broad representations of the features of animate creatures. Indeed, visually naïve chicks and monkeys have been found to extend their early social preferences to animals belonging to other species, even including potential predators (e.g. [5,14,34]). This suggests that recognition of conspecifics is most likely achieved by subsequent learning processes, like filial imprinting.

The initial works showing domestic chicks’ preferences for stuffed junglefowl hens attributed chicks’ preferences to the configuration of features that were present in the head of the stuffed hens (and of other stuffed animals as well, such as polecats and ducks, [7]). This prompted similar research in newborn babies, revealing a bias to look at schematic face-like stimuli and resulting in the development of the “CONSPEC and CONLERN” model [35], which has been very influential in the field of social developmental psychology (see for recent evidence in developmental neuroscience [36]).

Subsequent research in domestic chicks have further extended the parallelism between research in domestic chicks and human newborns, revealing similar preferences for schematic face-like configurations in visually naïve chicks [8,37].

Given the seminal role of chicks’ preferences for taxidermized jungle fowl hens in the development of this field (e.g., for recent neuroanatomical studies capitalising on this phenomenon see [38]), in the current study we decided to further explore this phenomenon, by addressing specific experimental question that had been left unanswered in the original studies. One of the most interesting claims originated from the initial works of Horn and his collaborators was that the preferences observed for the jungle fowl hen were due to the configuration of features characterising its head region. Moreover, this preference may not be selective for conspecifics, extending also to the head regions of other bird species or even mammalian predators [7]. This is in line with the adaptive functions of social predispositions and also with the subsequently obtained evidence that domestic chicks’ preferences for animate motion and face-like structures can be observed with stimuli representing other species (e.g. a human face [34] and the walking pattern of a cat [14]). However, in the original studies this conclusion was actually supported mostly by indirect or negative evidence. Specifically, [7] drew this conclusion based on the fact that they could not observe any preference between a “canonical” stuffed jungle fowl hen and a stimulus presenting only the head of a similar hen mounted over a box. Also, no preference was reported between the “canonical” fowls and scrambled stimuli in created reassembling the main body parts of another hen in unnatural configurations (in which the head and neck region and other big body parts were preserved in its integrity, even though displaced). Likewise, no preference emerged between the jungle fowl hen and a female specimen of gadwall duck, or between the fowl hen and a polecat. On the contrary, the “canonical hen” was preferred over the so called “texture hen”, created by cutting the pelt of a hen in very small pieces, attached in random order over a box. The “texture hen” had the same visual texture, colour and all the same local features, as the “canonical hen”, but the configuration of features characterising the head region, as well as any other major body parts, had been disrupted. However, no direct evidence has been provided so far on the presence of active preferences for taxidermized specimens belonging to other avian or mammalian species and on the role of the head region in determining these preferences. Moreover, contrary to what is the case for schematic face-like stimuli, the orientation of the head of the jungle fowl hen did not seem to be crucial for chicks’ preferences in the original works of Johnson and Horn ([7] Exp. 2.1), calling for further investigations on the role of the head region in chicks’ preferences for taxidermized animals. Another interesting aspect that has been overlooked by previous studies is the role of other chicks as social attachment objects. In fact, filial imprinting in chicks can occur not only towards the mother hen, but also towards siblings of the same batch, without impairing the brood cohesion. Indeed, in ducklings visual experience and interaction with siblings crucially affect the development of imprinting learning about the features of a maternal model [39–41].

In the current study, we wanted to further explore the inborn preferences of visually naïve domestic chicks for taxidermized animals. The first experiment represents a replication of previous studies [7,13,38,42] showing that, 24h after a non-specific stimulation procedure, visually naïve domestic chicks display a preference for approaching a stuffed fowl hen over a scrambled version of the same stimulus. The aim of this replication, which highlights the robustness of the phenomena under investigation, was to validate the procedure used in the following experiments. Moreover, the results of this experiment also provide a reference point to which to compare the performance of the chicks in the following experiments. In the subsequent experiments, we verified if an analogous preference could be observed also for a taxidermized chick compared to a scrambled chick specimen and we directly tested the presence of preferences for taxidermized specimens whose head region is visible compared to similar ones in which the head region is occluded. In order to clarify whether chicks’ social predispositions are sensitive to species-specific information, we employed for our stimuli specimens of females of another avian species (the mallard duck, *Anas platyrhynchos*) and of a mammalian predator species, the polecat (*Mustela putorius furo*).

## Material and methods

### Subjects, hatching and rearing conditions

Chicks (*Gallus gallus domesticus*) of the Ross 308 strain were used. Twelve chicks (of which 7 males) were used for the first experiment, 10 chicks (7 males) for Experiment 2, 11 (6 males) for Experiment 3 and 14 for Experiment 4, of which 9 were females and 4 males (information about the sex of one additional chick was lost; this subject was excluded from the analysis on sex differences). The sample of these experiments, although relatively small, is in line with that of previous studies that investigated the predisposition for the stuffed hen after acoustical priming (e.g., [43,44]). Here we decided to set the minimum sample size acceptable for each experiment at N = 10 (above that the precise sample depended on the number of chicks available in each batch). The sex of each chick was determined thanks to a sexual dimorphism in the feathers on the outer blade of the wing, at the time of hatching. Female chicks can be easily recognised because the primary feathers are longer than the covert feathers above them (males present cover and primary feathers of the same length or covert feathers longer than primary feathers). Sexing was done immediately after the end of the test, by gently spreading the wing of each animal and observing the length of the two rows of feathers.

Fertilised eggs were obtained from a local commercial hatchery (Agricola Berica, Montegalda (VI), Italy) and were hatched in total darkness. Hatching took place at a temperature of 37.7°C, with 60% humidity. To estimate approximate hatching time, each incubator was equipped with an infrared LED lamp and a camera (CCD Board camera 8.47mm, 1/3”). Photos were captured digitally every 20min with a custom-built time-lapse software.

Approximately 24h after hatching chicks were positioned in individual cardboard compartments (10×10cm), inside a dark incubator (33°C). All handling and transportation of the chicks occurred in the dark. After being placed in the individual compartments, chicks underwent an acoustic stimulation procedure, in order to elicit the subsequent expression of the predispositions. This procedure has been introduced by [43] and employed in our previous studies [13,38,42,44], in order to elicit the subsequent expression of the innate preference to approach the stuffed hen. Previous literature (reviewed also in [3]) revealed that this predisposition emerges only if chicks have been previously exposed to an unspecific stimulation (e.g., acoustical stimulation, motor activity, handling) during a sensitive period (occurring around 24h after hatching). The stimulation is defined “unspecific” since it does not provide to the chicks any information on the appearance of the hen. Thus it cannot provide the basis for any specific form of learning, but it is believed to alter the chicks’ physiological state (e.g. arousal level), allowing the subsequent expression of the predisposition. The stimulation (four 45-min sessions with 15-min intervals between sessions) occurred inside the individual compartments in the dark incubator, which was equipped with a loudspeaker. Non-species-specific sound stimulation was provided using a digitally constructed audio file composed of non-repeating rhythmic segments of music (see [38] for details).

### Test apparatus

The preference test was performed in a black rectangular arena, with a running wheel at the centre. For Experiments 1, 2 and 4 the arena was 150cm long, 60cm wide and 50cm high, and the running wheel was 7cm wide on the inside and 25cm in diameter (custom made for domestic chicks by TSE Systems, Germany). For Experiment 3 the arena was 150cm long, 46cm wide and 45cm high and the running wheel was 9.5cm wide on the inside and 33cm in diameter. Both setups had been already validated in our own previous studies on social predispositions for stuffed hens [13,42]. Two test stimuli (a target stimulus and a control stimulus) were placed separately on opposite ends of the corridor on two rotating platforms (20 rpm). Both stimuli were illuminated from above (40W warm light that diffused through a semi-transparent white plastic sheet). Additional illumination was provided by top/front lights (25W warm light), while the rest of the testing room was only dimly illuminated. From inside the running wheel chicks could see both ends of the corridor.

### Test stimuli

#### Experiment 1

**Fig 1:**
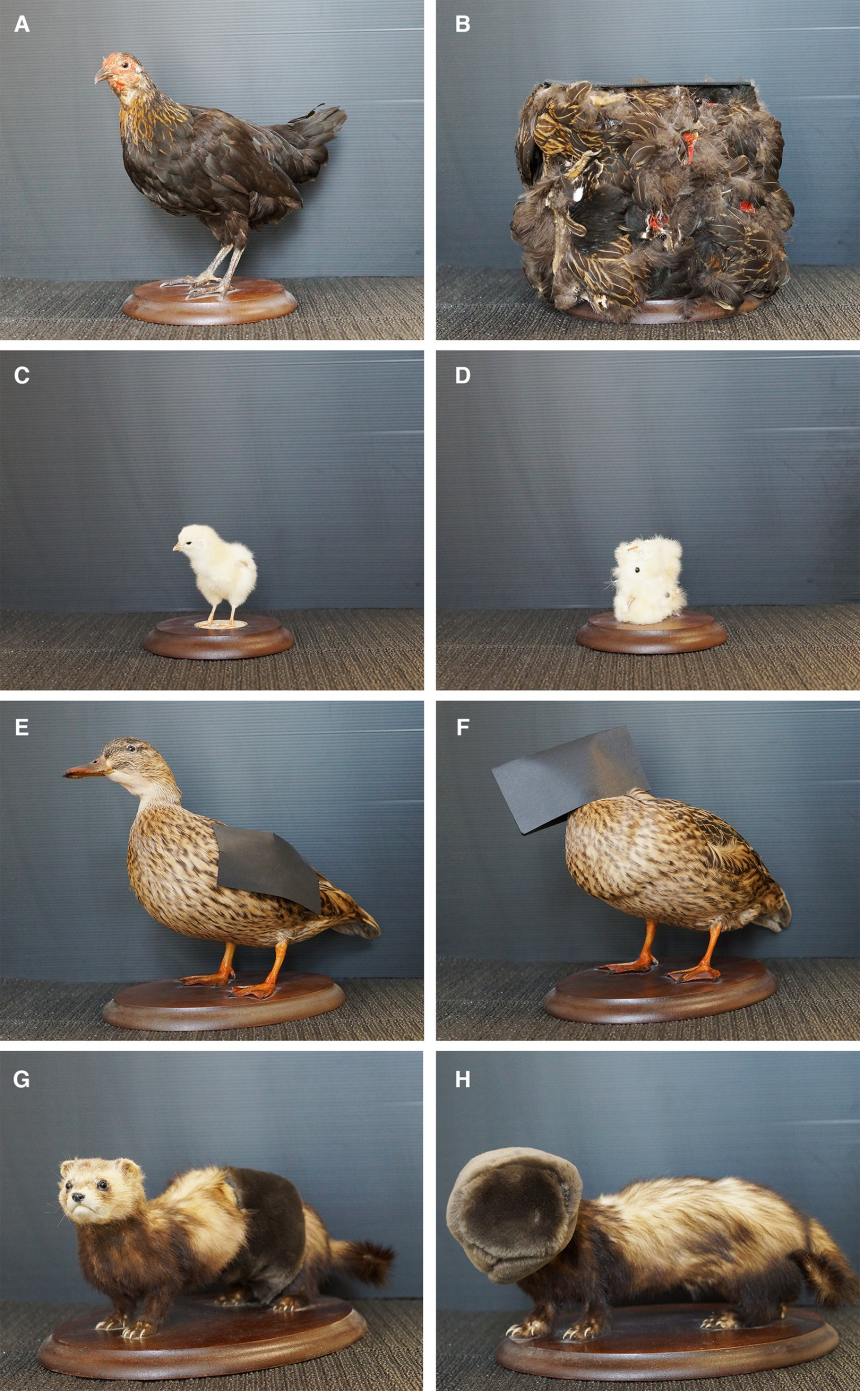
Test stimuli. Target stimuli (on the left, a, c, e, g) and control stimuli (on the right, b, d, f, h) used in Exp. 1 (a, b), Exp. 2 (c, d), Exp. 3 (e, f) and Exp. 4 (g, h).

The target stimulus used for Experiment 1 was the so called ‘stuffed fowl’, a taxidermized hen acquired from a local taxidermist and chosen to resemble the jungle fowl hen used also in the classical studies on chicks’ social predispositions [7]. The control stimulus was the so called ‘texture fowl’, obtained by a cutting in small pieces a second taxidermized hen and affixing them in a scrambled fashion to the sides of a box (Fig 1A and 1B). The maximum dimension of the ‘stuffed fowl’ was approximately 22cm in height, 25cm in length and 8cm in width, whereas for the ‘texture fowl’ the maximum dimension was 18.5cm in height, 26cm in length and 15cm in width. These two stimuli differed in their configuration, while being balanced for other visual properties such as local visual features luminance, colour, texture and movement. Similar stimuli were already employed in our previous studies [13,38,42].

#### Experiment 2

A ‘stuffed chick’ (a taxidermized chick of a similar looking breed as the experimental subjects) was used as a target stimulus. The control stimulus was a ‘texture chick’, obtained, as described in the first experiment, from another taxidermized specimen (Fig 1C and 1D). The maximum dimension of the ‘stuffed chick’ was 8cm in height, 8cm in length and 4cm in width, whereas for the ‘texture chick’ the maximum dimension was approximately 6cm in height, 4cm in length and 3cm in width. As for the first experiment, these two stimuli differed in their configuration, while being balanced for local visual features, luminance, colour, texture and movement.

#### Experiment 3

In this case, the target stimulus was a taxidermized mallard duck (*Anas platyrhynchos*), whose wings had been partially covered by two rectangular pieces of black cardboard (7x13.5cm), one on each side of the body, called the ‘no-wings duck’. The control stimulus was a similar duck specimen, whose head region had been covered by two rectangular pieces of black cardboard (one on each side of the head, 7x13.5cm), called the ‘no-head duck’ (Fig 1E and 1F). The maximum dimension of the ‘no-wings duck’ was approximately 25.5cm in height, 38cm in length and 14cm in width, whereas for the ‘no-head duck’ the maximum dimension was 24.5cm in height, 38cm in length and 12cm in width. Please note that to create the stimuli for this experiment we used ducks belonging to a different species than those employed by Johnson and Horn [7], who employed two gadwall ducks (*Anas Strepera*). This was due to technical problems in obtaining gadwall duck specimens for the current study.

#### Experiment 4

In the last experiment, the target stimulus was a taxidermized polecat specimen (*Mustela putorius furo*), whose trunk had been partially covered by a stripe of artificial brown pelt (7cm wide), the ‘no-trunk polecat’. The control stimulus, the ‘no-head polecat’, was obtained covering the head region of a similar specimen, by inserting it in a cylinder of the same artificial pelt (7cm wide) (Fig 1G and 1H). The maximum dimension of the ‘no-trunk polecat’ was approximately 14cm in height, 34cm in length and 13.5cm in width, whereas for the ‘no-head polecat’ the maximum dimension was 16.5cm in height, 35.5cm in length and 16.5cm in width.

### Test procedure

Approximately 48h after hatching, chicks underwent a free choice test, in which they expressed the preference between a target and a control stimulus. A different pair of stimuli, obtained from taxidermized animals, was used in each experiment (see above for details). For each pair of stimuli, in the target stimulus the head and face of the animal were clearly recognisable. In the control stimulus, on the contrary, the head region had been scrambled or occluded. Each chick participated only to one experiment and was tested only once. Before the test, chicks were constantly maintained in darkness, so that they were visually naive at the time of test.

To test the spontaneous preference between the target stimulus and the control stimulus, each individual chick was placed in the running wheel facing the wall, so that at the start both stimuli were equally visible (chicks have laterally placed eyes and a wide visual field). The left-right placement of the stimuli in the corridor were counterbalanced between subjects. Chicks could make the wheel rotate by running towards a given stimulus and could also easily move inside the wheel to alternate their direction of running. The test duration was 30min. During the test chicks were recorded with a video camera. The number of rotations of the wheel in each direction was counted by an automated system integrated into the wheel and the sums of rotations made in each direction were displayed on a small digital screen.

### Statistical analysis

The total number of rotations made towards the target stimulus and the control stimulus was used to calculate the preference score for the stuffed fowl for each individual chick (*preference score*: number of rotations towards target/total number of rotations). Values of this score could range from 0 to 1, with a value of 1 indicating an exclusive choice (i.e., preference) for the target stimulus and a value of 0 indicating preference for the control stimulus. To analyse group performance, a one sample two tailed t-test was used to reveal significant departures from chance level (corresponding to a preference score of 0.5). Moreover, each subject was categorised as having an absolute preference for either the target or the control stimulus, based on the values of the preference scores (individuals who run greater distances towards the target stimulus had score values equal or higher than 0.501, whereas individuals who run more towards the control stimulus had score values equal or lower than 0.499). The frequency distribution of these individual values was compared against chance by a chi-square test. A t-test for two independent samples was used to compare the preference score of male and female chicks and between different experiments (the Levene test was used to test the homogeneity of variances; no violations were detected). All tests were two-tailed. Statistical analysis was performed with the software IBM SPSS Statistics for windows version 24.0 and G*Power 3.1.9.4 [45].

### Ethical statement

The experiments reported here comply with the current Italian and European Community laws for the ethical treatment of animals. All the experiments and experimental procedures were licensed by the Ministero della Salute, Dipartimento Alimenti, Nutrizione e Sanità Pubblica Veterinaria (permit number 20269/A).

## Results

### Experiment 1

**Fig 2:**
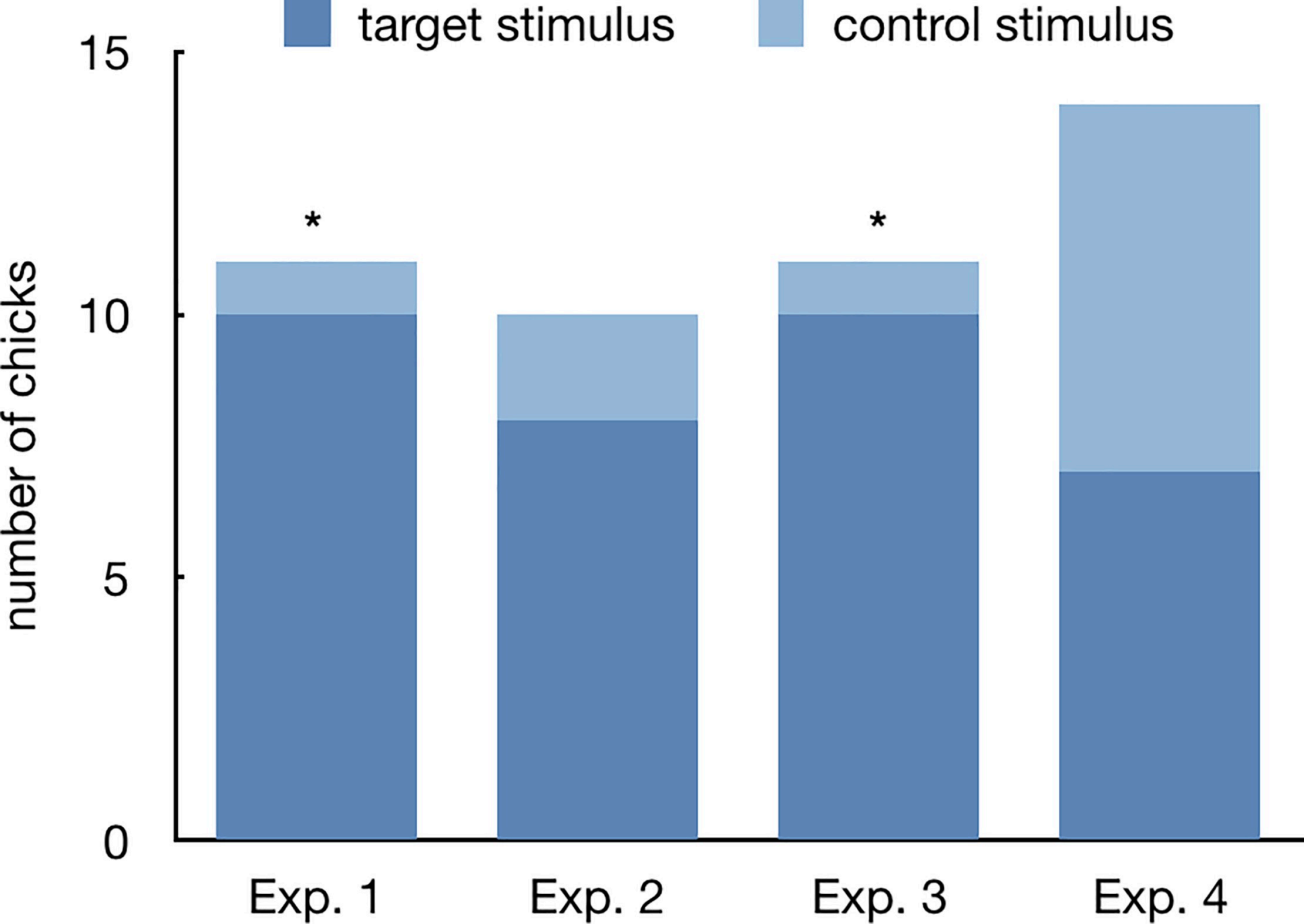
Absolute preference. Number of chicks (Y axis) showing an absolute preference for the target or the control stimulus (in dark and light blue, respectively), plotted for each experiment. Asterisks (*) represent significant departures from a random distribution (p<0.05).

**Fig 3:**
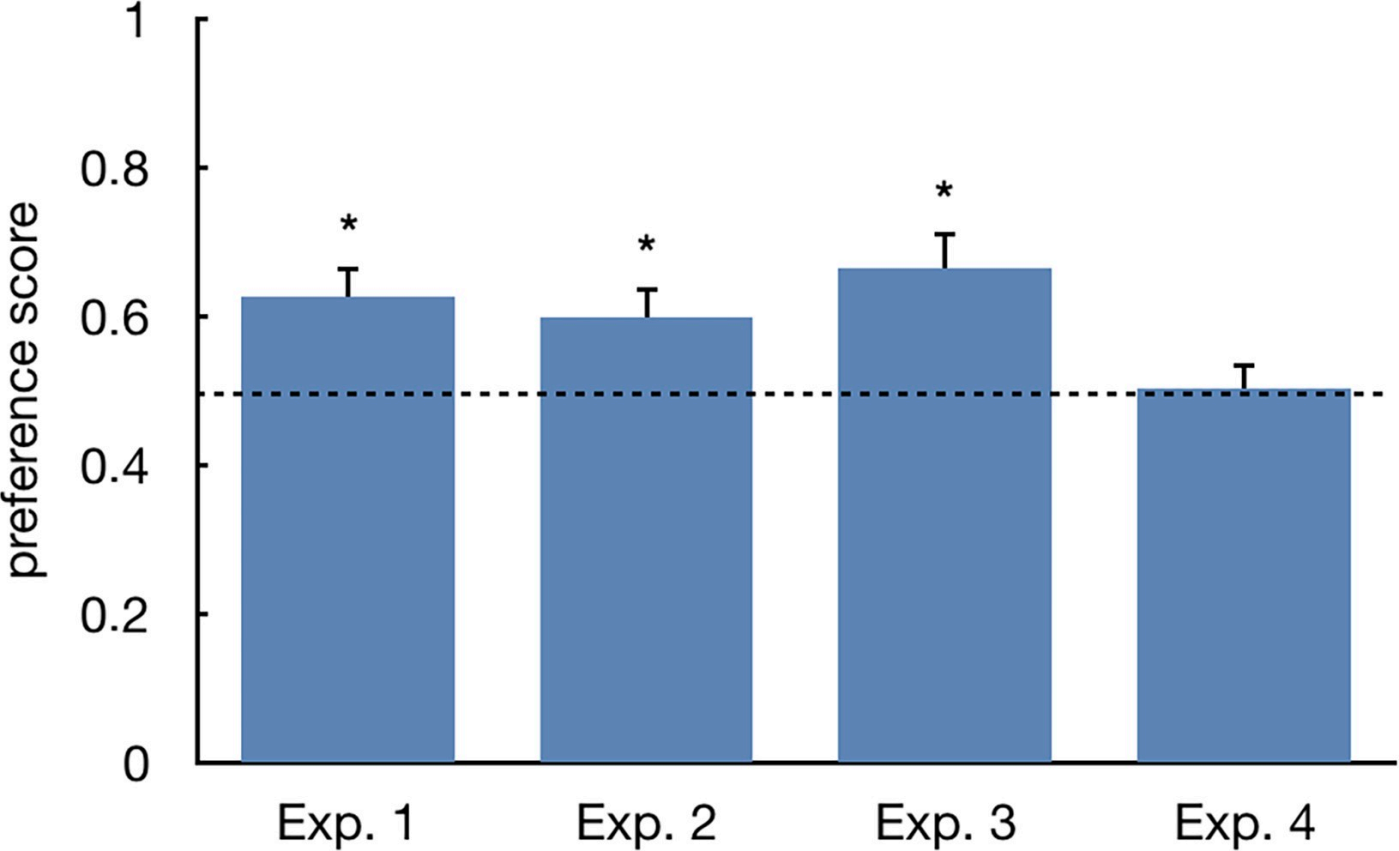
Preference score. Average preference score (Y axis) for each experiment. Means and S.E.M. are shown. Asterisks (*) represent significant departures from the chance level of 0.5, marked by the dotted line (p<0.05).

In the first experiment chicks covered an average of 4685.4±1275.1cm during the test. Ten out of 12 subjects showed an absolute preference in favour of the ‘stuffed fowl’ stimulus and one subject showed an absolute preference towards the ‘texture fowl’ (X^2^ = 7.374, p = 0.012) (one individual was excluded from this analysis because it run exactly the same distance towards either stimulus) (Fig 2). Also the group analysis of the preference score revealed a significant preference for approaching the ‘stuffed fowl’ (mean±SEM = 0.626±0.044, t_11_ = 2.874, p = 0.015, d = 0.830, 95% confidence interval of the difference 0.029 to 0.22; Fig 3). The level of preference observed was not different between the sexes (males 0.589±0.044, females 0.678±0.086, t_10_ = -0.999, p = 0.341, d = 0.226, 95% confidence interval of the difference -0.286 to 0.109).

### Experiment 2

Chicks covered an average of 3659.3±655.7cm during the test. Eight out of 10 subjects showed an absolute preference in favour of the ‘stuffed chick’ stimulus and two showed an absolute preference towards the ‘texture chick’ (X^2^ = 3.600, p = 0.109, Fig 2). The group analysis of the preference score revealed a significant preference for approaching the ‘stuffed chick’ (0.599±0.04, t_9_ = 2.493, p = 0.034, d = 0.788, 95% confidence interval of the difference 0.009 to 0.189; Fig 3), which was not different between the sexes (males 0.581±0.051, females 0.640±0.066, t_8_ = -0.654, p = 0.531, d = 0.166, 95% confidence interval of the difference -0.265 to 0.148).

### Experiment 3

In the third experiment chicks covered an average of 7764.3±2481.9cm walking inside the wheel. Ten out of 11 animals showed an absolute preference towards the ‘no-wings duck’ (X^2^ = 7.364, p = 0.012; Fig 2). Likewise, the group analysis of the preference score revealed a significant preference for approaching the ‘no-wings duck’ (0.664±0.050, t_10_ = 3.267, p = 0.008, d = 0.985, 95% confidence interval of the difference 0.052 to 0.276; Fig 3). Once again, the choice between the two stimuli was not different between the two sexes (males 0.649±0.078, females 0.683±0.068, t_9_ = -0.325, p = 0.753, d = 0.083, 95% confidence interval of the difference -0.274 to 0.205).

### Experiment 4

During the test chicks covered an average of 5474±1879.6cm walking in the wheel. Seven out of 14 chicks showed an absolute preference towards the ‘no-trunk polecat (X^2^ = 0.000, p = 1.000, Fig 2). Likewise, the group analysis of the preference score did not reveal any preference between the two stimuli (mean±SEM = 0.502±0.035, t_13_ = 0.062, p = 0.951, d = 0.016, 95% confidence interval of the difference -0.072 to 0.077, Fig 3). No sex differences were apparent (males 0.455±0.037, females 0.521±0.051, t_11_ = -0.809, p = 0.436, d = 0.197, 95% confidence interval of the difference -0.246 to 0.114).

### Between-experiments comparison

We also wanted to compare the preferences observed for the different stimuli to the results obtained with the ‘stuffed-fowl’ and ‘texture-fowl’. This pair of stimuli, for which a robust effect is reported in the literature, provides a useful point to evaluate the entity of the preference elicited by the new stimuli tested. An ANOVA on the ratio of preference (two between subjects factors: *stimuli*, four levels; *sex*, two levels) revealed a significant difference between the four experiments (main effect of *stimuli*, F_(3,38)_ = 2.978, p = 0.042; no other significant effect or interaction emerged, *sex* F_(1,38)_ = 1.789, p = 0.189; sex*stimuli F_(1,38)_ = 0.063, p = 0.979). No significant difference emerged between the ratio of preference for the target stimulus in Experiment 1 and 2 (t_20_ = 0.445, p = 0.661, d = 0.139, 95% confidence interval of the difference -0.099 to 0.152), or in Experiment 1 and 3 (t_21_ = -0.580, p = 0.568, d = 0.097, 95% confidence interval of the difference -0.177 to 0.1), indicating that the level of preference for the ‘stuffed chick’ and the ‘no-wings duck’ was similar to that elicited by the ‘stuffed fowl’. On the contrary, a significant difference emerged between Experiment 1 and 4 (t_24_ = 2.248, p = 0.034, d = 0.330, 95% confidence interval of the difference 0.010 to 0.237), indicating, as expected that the level of preference for the ‘no-trunk polecat’ was lower than that for the ‘stuffed fowl’.

## Discussion

The aim of this study was to investigate the early emerging preferences that visually naïve domestic chicks display for taxidermized animals. This phenomenon has been known in the literature for three decades (e.g., [7]) and has provided the basis for the development of seminal theories, with great influence on various research fields, from developmental psychology to comparative cognition [3,6,35,46,47]. Here, we further clarify the mechanisms underlying chicks’ preferences, focusing on the role of the head region of models of different ages and species. By demonstrating that the duck stimulus whose wings have been covered is preferred over a similar stimulus whose head region has been covered (Exp. 3), we provide the first direct evidence that the visibility of the head region of taxidermized models drive chicks’ approach preference in this classical test. Since the two stimuli were well matched in their size, general appearance and in the extent of body surface occluded, the preference expressed by chicks in the running wheel test cannot reasonably be attributed to properties other than the visibility of the head region of the taxidermized animal. Interestingly, this result has been obtained using taxidermized specimens of mallard duck (*Anas platyrhynchos*). This confirms the original hypothesis put forward by [7]: that the attraction for the head region indeed extends to females of other species, rather than being limited to hens of the *Gallus gallus domesticus*.

Moreover, we have also found a preference for a canonical taxidermized domestic chick, over a severely scrambled version of the same stimulus (texture chick), whose body structure was completely disrupted (Exp. 2). This shows that social predispositions can be guided by a configuration of features recognizable in the canonical body structure of young chicks (probably localised in the head region), extending to same-age individuals the results that have been previously obtained with taxidermized hens [7,13,38,42]. Even though scholars working with domestic chicks have since long known that chicks easily develop social attachment to each other (e.g., see [48] for a review), this is the first demonstration that chicks’ predispositions can direct attention also on their siblings, and not only on the mother hen. This is likely to have positive consequences for adaptation, since attraction and attention toward other chicks of the brood will be effective in maintaining the brood cohesion, while contact with other chicks also offers opportunities for thermal regulation in the absence of the mother hen. Moreover, domestic chickens are a social species living in stable groups characterised by a dominance hierarchy (the pecking order) and social learning capabilities, which emerge shortly after hatching [49–51]. Mechanisms directing attention towards other hatchlings could have an important role in the early development of social hierarchies, social learning by observation and other adaptations for social life. Indeed, research in ducklings has revealed a complex interaction between preferences for, and imprinting on, the mother and the siblings. Visually-naïve mallard ducklings do not show any preference between a taxidermized adult female duck and taxidermized ducklings, while, after imprinting on each-other, socially reared ducklings start to prefer the taxidermized ducklings to the adult female [40]. Thus, at least in this species, fellow hatchlings could be not less attractive than the mother as a potential imprinting object. On the other hand, moderate (but not prolonged) exposure to live ducklings has been reported to increase imprinting learning towards a taxidermized female duck ([39,41], [31] for a review). This suggests the presence of intricate relationships between the effects of preferences for, and consequent exposure to, the brood mates and the mother, which should be further explored also in domestic chicks.

Interestingly, the degree of preference displayed by the subjects for the taxidermized chick (Exp. 2) and for the taxidermized duck (Exp. 3), was similar to that observed in Exp. 1, in which we used the standard pair of stimuli comprising the canonical hen and its severely scrambled version, the texture hen. The lack of significant differences between these experiments should of course be interpreted with caution. However this could support the hypothesis that chicks’ preferences in the first three experiments are driven by the action of the same mechanism, likely a preference for the configuration of features present in the head region of the specimens [8].

Contrary to what was the case for the first three experiments, in Exp. 4 we were unable to demonstrate any preference for the polecat whose head was visible over the one whose head was covered. The comparison between the data of Exp. 1 and 4 confirmed the difference in chicks’ behaviour between the two studies. This is in conflict with our initial predictions, because, even though polecats are a mammalian species and a potential predator to domestic chickens, early social predispositions are usually considered non-species specific. Indeed, previous evidence revealed that unlearned preferences for biological motion extend to the walking pattern of domestic cats [14]. Likewise, preferences for two-dimensional images of faces extend to photographic images of human faces [34]. Finally, in the original study by [7], chicks did not display any preference between a taxidermized hen and a polecat, in apparent conflict with our current results. This apparent contradiction could be attributed to the several methodological differences occurring between the current study and the earlier work by Johnson and Horn. Among these differences there are for example the kind of stimulation procedure employed to elicit the emergence of the predisposition ([7] employed motoric activity as a stimulating experience, whereas in the current study we used acoustical stimulation, after [43]; see also [13,38]) and the different strain of chicks used (it has been found, for example, that chicks of various strains differ in the degree of preference for the canonical hen, [42]). The most notable difference between our procedure and that of the original study by [7] is probably the pair of stimuli used at test. While we directly compared chicks’ preference for a polecat whose head region is visible and one with the head covered, Johnson and Horn compared the polecat to the canonical hen, without finding any preference between the two. Since their work did not contain any direct evidence of the preference for the head region of the polecat, the data gathered in these studies are not necessarily in conflict with each other.

Regardless of the methodological comparison with previous works, one possible interpretation of the lack of preference observed in Exp. 4 could be the presence of antipredatory responses, activated by the polecat stimulus, which could have conflicted with the preference for the head region, blocking its expression. It is tempting to speculate that the attractiveness of the stuffed polecat predator stimulus, reported by [7], could be rooted in different mechanisms than the predisposition favouring attention towards the head region, which seem to cause the preference for conspecifics and other birds. The predator/prey relationship between chicks and natural predators may be subject to an arms race in which the predator has evolved characteristics to make itself attractive to the prey, by means of a mechanism other than the facial predisposition.

On this regard, however, it is also worth noting that ducks might readily attack domestic chicks (even though probably much less frequently than mammalian predators). Indeed, even mallard ducks have been recently documented to prey on small birds [52]. Nevertheless, as we have already mentioned before, non-significant results should be interpreted with particular caution, since there are a number of practical and methodological reasons that could explain a failure to demonstrate a preference for the polecat stimulus. The issue of whether chicks’ preferences for the head region of taxidermized animals can extend also to mammalian predator species should thus be considered still unresolved, until further clarified by future studies.

Another issue that could be investigate by subsequent studies is the presence of gender differences in chicks’ predispositions for static configurations of features. Previous literature reported gender differences, when the animals were tested with two-dimensional face-like stimuli containing eye-like concentric features [37]. It is also well known that female chicks have stronger motivation for social reinstatement than males [53]. In the current study we have been unable to demonstrate any significant difference in the level of preference exhibited by males and females. However, in all the experiments, a similar trend consistently emerged, with females having higher levels of preference than males (for instance, this was particularly pronounced in the first experiment). This would be in line with what was found for face-like stimuli with concentric eye features. These stimuli elicited relatively stronger approach responses in females, whereas males tended to avoid them, possibly due to the activation of antipredatory mechanisms in response to the eye-like shapes. Future studies could thus further explore the presence of gender difference in the responses to naturalistic stimuli, such as the taxidermized specimens we employed here. This could be done, for example, by using stimuli whose eyes present a conspicuous concentric organization, with clear contrast between the iris and the pupil. This feature was absent in our stimuli, that had uniformly black eyes (see Fig 1) and could be relevant to elicit strong differences in the responses of male and female subjects, allowing the study of the interplay of affiliative and antipredation responses in the two sexes.

## Supporting information

S1 Dataset Original data of the four experiments. (XLSX)
